# Gastrointestinal stromal tumor: an incidental finding during laparoscopic bariatric surgery

**DOI:** 10.1002/ccr3.1194

**Published:** 2017-09-25

**Authors:** Shireesh Saurabh

**Affiliations:** ^1^ Mercy Hospital 540 East Jefferson Street, Suite 205 Iowa City 52245 Iowa

**Keywords:** Bariatric surgery, gastrointestinal stromal tumor, laparoscopic sleeve gastrectomy, morbid obesity

## Abstract

Gastrointestinal stromal tumor (GIST) is the most common mesenchymal tumor of the gastrointestinal tract. Lack of clinical symptoms and findings on preoperative upper endoscopy makes its diagnosis difficult in bariatric patients. A laparoscopic resection of the gastric GIST during bariatric surgery is associated with good long‐term prognosis.

Question: Fifty‐one‐year‐old female with obesity (body mass index 38.2 kg/m^2^), hypertension, and sleep apnea underwent an elective laparoscopic sleeve gastrectomy. She had no symptoms suggesting the presence of a gastric tumor and preoperative upper endoscopy was normal. During surgery, a 1 cm nodule was found on the proximal anterior surface of the stomach (Fig. 1). It was resected along with the gastric specimen, and histopathology showed spindle‐shaped cells (Fig. 2). What is the diagnosis?

**Figure 1:**
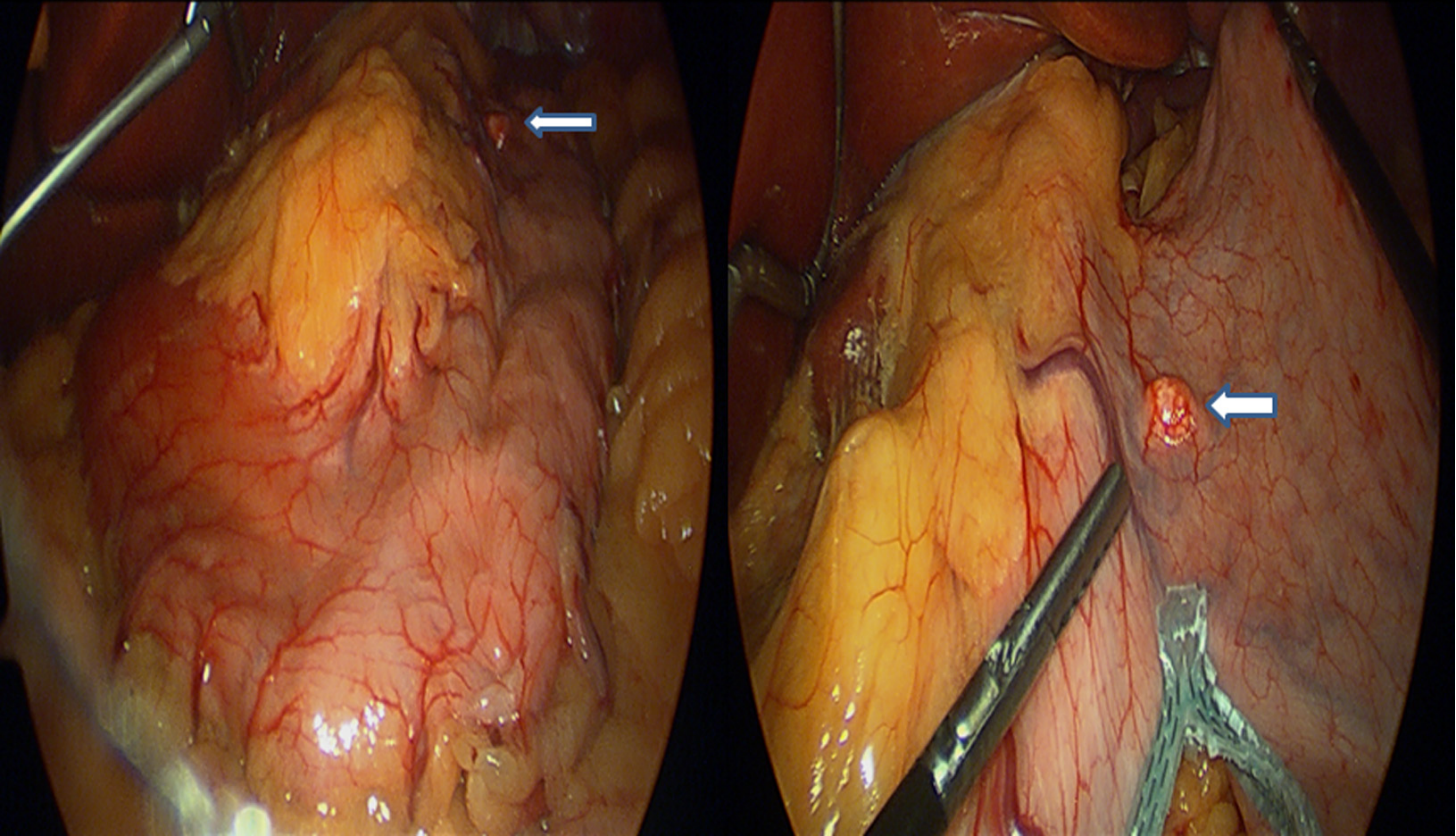
Intraoperative picture during laparoscopic sleeve gastrectomy demonstrates gastrointestinal stromal tumor (white arrow) on the anterior surface of the stomach.

**Figure 2:**
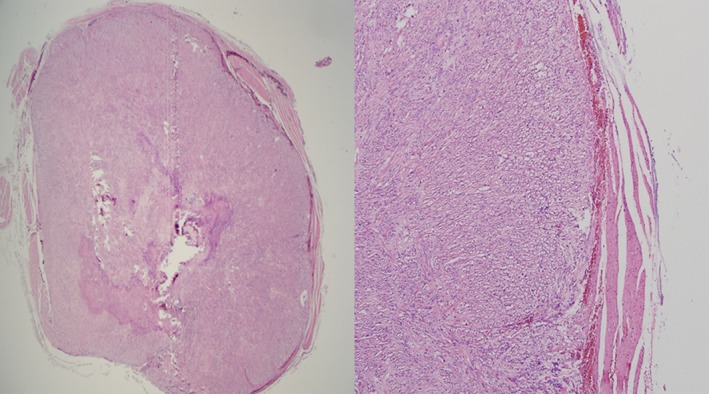
Microscopic image demonstrating spindle‐shaped cells on histopathology.

Answer: Gastrointestinal stromal tumor (GIST) of stomach.

The incidence of GIST is higher in obese patients 1. Symptoms are nonspecific, which makes the diagnosis difficult. Most GIST in bariatric surgery patients are incidental intraoperative findings and usually have low‐grade malignancy 2. Surgery is the mainstay of therapy, and the goal was to achieve negative microscopic margins 1. Lymph node metastasis is rare. It can be managed by laparoscopic approach.

## Conflict of Interest

None declared.

## Authorship

SS: designed, acquired, analyzed, interpreted the data. Drafted and approved the work to be published.

## References

[1] Chiappetta, S. , S. Theodoridou , C. Stier , and R. A. Weiner . 2015 Incidental finding of GIST during obesity surgery. Obes. Surg. 25:579–583. 25596937 10.1007/s11695-015-1571-4

[2] Viscido, G. , F. Signorini , L. Navarro , M. Campazzo , P. Saleg , V. Gorodner , et al. 2017 Incidental finding of gastrointestinal stromal tumors during laparoscopic sleeve gastrectomy in obese patients. Obes. Surg. 27:2022–2025. 28185152 10.1007/s11695-017-2583-z

